# All-Inside Arthroscopic Meniscus Repair for Patients Over 40 Years of Age: Is Forty the New Twenty?

**DOI:** 10.7759/cureus.56413

**Published:** 2024-03-18

**Authors:** Gianluca Ciapini, Giorgio Varchetta, Federico Bizzocchi, Giulio Gadsby, Leonardo Lombardi, Francesca Sgadò, Edoardo Ipponi, Michelangelo Scaglione, Paolo Domenico Parchi

**Affiliations:** 1 Department of Orthopedics and Traumatology, University of Pisa, Pisa, ITA

**Keywords:** level of motor functionality, meniscal suture, older patient, meniscal tear, all-inside repair

## Abstract

Introduction: Meniscal injuries are a common challenge in orthopedic surgery. For decades, partial or total meniscectomy has been the primary surgical treatment for meniscal tears. In recent years, the increased recognition of menisci’s biomechanical importance has progressively shifted the paradigm towards meniscus repair. However, meniscus-sparing surgery remains the treatment of choice for selected lesions in young and active patients, especially for young and active patients. In this study, we evaluated the effectiveness of all-inside sutures in treating meniscus tears in patients over 40.

Methods: In our retrospective evaluation, we evaluated the clinical and functional outcomes of cases over 40 years of age with post-traumatic acute meniscus tears that received meniscus repairs using the all-inside technique. The pre-operative and post-operative functionality of treated patients were assessed using the knee injury and osteoarthritis outcome score (KOOS) score. Major complications were recorded.

Results: Twenty-three cases met our inclusion criteria. Eight females and fifteen males with a mean age of 44.9 were included in our study. Their mean follow-up was 35.1 months. Before surgery, our patients’ mean KOOS score was 55.4 (18-80). At the patients’ latest follow-up, the value had risen to 87.4 (63-100). There was no statistical correlation between patients’ age and their functional recovery. No surgical failure was recorded at the latest follow-up.

Conclusion: The all-inside suture technique can represent a suitable and reliable solution for suturable meniscal tears, even for patients over 40. Preserving the meniscus and restoring patients’ functionality allows patients to return to their daily activities and promote their quality of life.

## Introduction

Meniscal injuries are common, with an overall incidence rate (IR) of 8.3 per 1000 person-years. Men have an almost 20% higher risk than women of sustaining at least one such injury in their lifetime [1]. Meniscal tears can cause the onset of knee instability, pain, and eventually articular blocks, impeding patients’ daily living activities and harming their quality of life [2]. For decades, partial or total meniscectomy has been the primary surgical treatment for meniscal tears [2,3]. However, the increased recognition of the biomechanical importance of the menisci has shifted the paradigm towards preservation surgery, especially in younger and more active patients [2,3]. The menisci increase the stability of the femorotibial articulation, distribute the axial load, lubricate the knee joint, and have a role in the articular proprioception. Damage or surgical removal of the meniscus leads to degenerative changes within the tibiofemoral joint, exposing the knee to an increased risk of osteoarthritis. For these reasons, in recent years, meniscus spearing surgery, relying on meniscal sutures, has spread and has been established as a reliable solution for young and active patients [2,3]. Although the literature testifies that meniscal repair surgery often has good clinical and functional results for young patients [4,5], most surgeons are still reluctant to perform meniscus sutures for patients over 40 [2-5]. This reticence, mainly based on the higher degree of degeneration of the inner meniscus in older patients and the potentially reduced healing capacities of mid-aged patients, may collide with both the lesions’ epidemiology and patients’ demands. Epidemiological studies proved that meniscus tears are 2 to 4 times more likely to occur in patients over 40 years of age [1,6]. Furthermore, even for middle-aged and seniors, returning to routine sports and higher activity levels could be pivotal in pursuing patients’ physical and mental health [7,8]. For these reasons, extending the meniscus-sparing surgery to over 40 patients could theoretically allow a larger share of Western countries’ population to stay active and healthy in mid- to long-term scenarios.

In this article, we evaluated the clinical and functional effectiveness of arthroscopic all-inside meniscal sutures to treat meniscus tears in patients over 40 years of age.

## Materials and methods

This single-center retrospective study was performed following the ethical standards of the 1964 Declaration of Helsinki and its later amendments.

Our study consisted of a review of all the patients over 40 years of age who had been diagnosed with meniscal tears of the knee and were treated with an arthroscopic approach using all-inside sutures in our institution between May 2018 and May 2023.

Inclusion criteria were (I) a recent trauma of the knee, (II) MRI evidence of meniscal tear, (III) later confirmed under arthroscopic evaluation, (IV) the involvement of the red-red or red-white area of the meniscus, and (V) the use of at least one all-inside suture to repair the lesion. Only cases with a score of 3 or higher according to the Tegner activity scale were treated and included in our study. Exclusion criteria were (I) being under 40 years of age at the moment of surgery, (II) a degenerative nature of treated lesions, (III) concomitant lesions whose presence or treatment could slow or impede the post-operative rehabilitation process, and (IV) a follow-up shorter than six months. Each case received surgical treatment within two months after their injury.

We recorded general data for each patient, including age, gender, and localization of the meniscal tear. Pre-operative MRI images were used to orient the pre-operative diagnostic process and orient the surgical approach of choice. The diagnosis was later confirmed intra-operatively with arthroscopic evaluation.

Each case underwent a careful clinical examination to assess the pre-operative clinical status of each patient and the performance of each injured knee. The pre-operative functionality was assessed using the knee injury and osteoarthritis outcome score (KOOS).

Surgical approach

**Figure 1:**
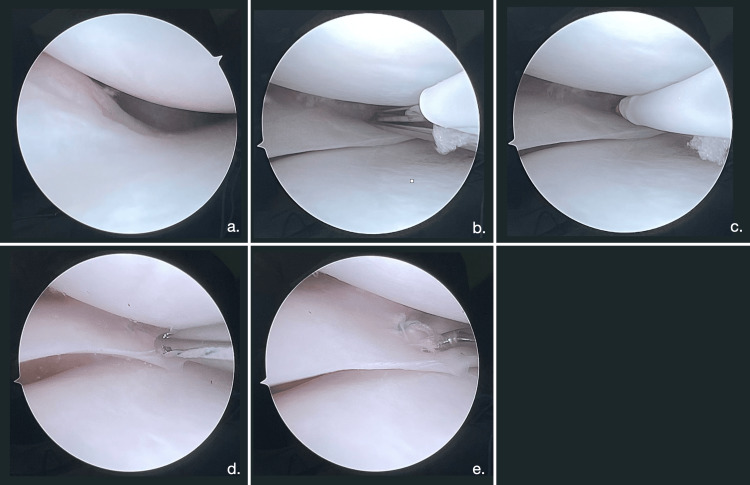
A ramp lesion reduced using an all-inside technique A ramp lesion is identified. (a) The metal slotted cannula is introduced into the joint with the needle. (b) The horizontal suture is made. (c) The knot is pushed and receded holding tension on the suture, then cut the suture. (d) A stability test is made with the probe. (e) This pictured meniscus belongs to a 43-year-old Caucasian male without previous knee surgeries or systemic comorbidities (case 4 in our cohort).

The patients were placed supine on the operative table, with the leg hanging off the table from the knee distally. A sterile field was prepared to cover the lower limb. Standard arthroscopic portals were made. A diagnostic evaluation of the articular cavity was carried out to identify the lesion and confirm that it was suitable for suturing. The torn meniscus was manipulated and reduced or cleaned (Figure 1) while a probe was used to measure the depth of the lesion. All cases were treated with all-inside sutures, using the Fast-FixTM 360 Meniscal Repair System (Smith & Nephew Endoscopy, Andover, US). The Fast-FixTM 360 delivery needle was introduced tip-down into the joint with the help of a slotted cannula that eases the passage through the fat pad, helps stabilize the meniscus, and maximizes the visualization of the area. The needle was inserted in the capsular side of the tear and the deployment slider was pushed all the way forward until a “click” sound was emitted to confirm the first implant was placed. The delivery needle was then slowly withdrawn from the capsular side of the tear and relocated to the inner meniscal fragment, at least 5 mm from the tear line. The second implant was set in place following the same process as described above. At this point, the suture was pulled to achieve a sliding knot and permanently reduce the tear. With the knot firmly in place and the wire kept under tension, the suture was finally cut with a 2-3 mm tail to end the repair. This procedure was performed once or repeated a second time, depending on the tear length. Intraoperative meniscal stability was evaluated before the end of the surgical procedure (Figure 1). Ending surgery, arthroscopic devices were removed, the portals were sutured, and the knee was wrapped in an elastic bandage.

Post-operative treatments and follow-up

During their hospitalization, the patients were daily assisted by our physiotherapists to start their rehabilitation. Free active and passive mobilization was allowed since the first day after surgery, avoiding passive mobilization over 90 degrees of flexion for the first 30 days after surgery. Progressive weight bearing was allowed within 15 days after surgery, with the scheduled goal to achieve the complete load on the treated limb 30 days after surgery.

The post-operative follow‐up consisted of periodic orthopedic office visits, with clinical evaluations. Each intra‐operative and post-operative complication, of grade II or higher according to the Clavien-Dindo classification, was included and mentioned in our study. The KOOS score was calculated for each patient at their latest follow-up, to allow a comparison between pre-operative and post-operative functionality.

## Results

Between May 2018 and May 2023, 23 over 40 patients were diagnosed with meniscal injuries of the knee and treated with meniscal repair using all-inside sutures. There were 8 females and 15 males. Their mean age was 44.9 (40-54). About 11 patients had a lesion in their left knee and 12 suffered from tears in their right knee. In 13 cases we treated the medial meniscus, and in 10 cases we treated the lateral meniscus.

Before surgery, our patients’ mean KOOS score was 55.4 (18-80). During surgery, none of our cases had major complications. The mean post-operative follow-up was 35.1 months (9-72). No surgical failure (defined as a necessity of reintervention for recurrence of the same clinical picture) was recorded at the latest follow-up. One of our cases, due to suspected septic arthritis, underwent arthroscopic washout and tissue sampling for microbiological and histological analysis. These examinations did not find any evidence of local infection and were oriented toward a diagnosis of idiopathic chronic inflammation. Despite this issue, the patient achieved a fair KOOS score (73). No other event or major complication was reported through the post-operative intercourse of the other patients. A schematic representation of our cohort is reported in Table 1.

**Table 1:** Schematic summary of our cohort M: Medial meniscus; L: Lateral meniscus; ♂: Male; ♂: Female; KOOS: Knee injury and osteoarthritis outcome score

Case	Age	Gender	Meniscus	KOOS pre	Stitches	KOOS post	Complications
1	45	♂	M	50	2	74	None
2	49	♀	L	18	1	89	None
3	41	♂	M	45	2	96	None
4	43	♂	M	70	2	99	None
5	42	♂	M	65	2	97	None
6	48	♂	M	60	2	96	None
7	42	♀	M	42	1	97	None
8	49	♀	M	55	1	88	None
9	49	♂	L	59	2	76	None
10	45	♂	L	80	2	85	None
11	54	♂	L	72	1	100	None
12	40	♀	M	16	1	100	None
13	41	♂	L	62	1	90	None
14	45	♀	M	70	2	83	None
15	40	♂	L	63	1	63	None
16	45	♂	L	60	1	85	None
17	44	♀	M	65	2	99	None
18	49	♀	L	60	1	85	None
19	43	♂	M	42	2	73	Chronic inflammation
20	46	♀	L	32	1	74	None
21	45	♂	M	50	1	74	None
22	49	♀	L	18	1	89	None
23	41	♂	M	45	1	96	None

**Figure 2:**
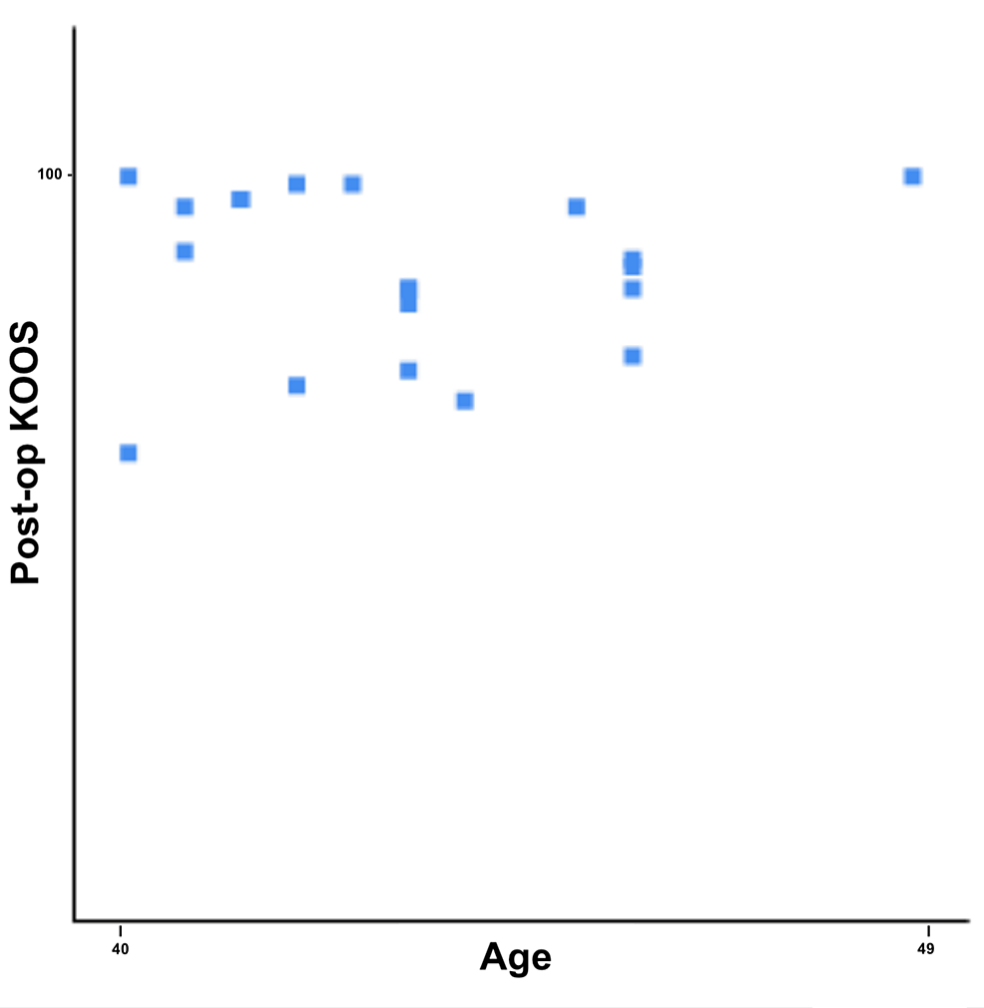
Graphic representation of post-operative KOOS distribution per patients’ age KOOS: Knee injury and osteoarthritis outcome score

The mean post-operative KOOS score of our cohort was 87.4 (63-100). This value was significantly higher than the pre-operative one, as testified by a two-tailed T-student test (p=0.0001). Both a Pearson correlation test and a Spearman correlation test were used to assess whether there was a correlation between age and functional outcomes (Figure 2). These tests could not find any statistically significant correlation between age and functional recovery (p=0.860 and p=0.584, respectively).

## Discussion

Meniscal tears represent a growing problem for the national healthcare systems worldwide. Since the first decade of the new millennium, epidemiological population studies showed that meniscus tears requiring treatment are two to three times more common in patients over 40 years than in those under 40 [6,9]. However, for years, the classical teaching in arthroscopic knee surgery has considered age as a relative patient-related contra-indication for meniscus sutures and repair. This line of reasoning is based on the fact that older patients are more likely to have concomitant degenerative cartilage changes, develop chronic tears, or suffer from unfavorable degenerative tear patterns such as horizontal cleavage tears [10-12].

Despite these theoretical risks, the correlation between patients’ age and the success of meniscal repair is still unclear. In modern literature, some studies reported that increased age was associated with higher failure risk [13,14], some suggested a lower failure risk [15-18], while others showed no association between age and complication rates [19]. In recent years, the concept of a ceiling age for patients’ eligibility for meniscal sutures has been progressively fading. In 2014, Mordecai et al. [20] proposed a flowchart for the surgical treatment of meniscal tears, including patients’ age, global clinical status, and tears’ localization. Their flowchart suggested meniscus repair for lesions localized in the red-red zone in patients up to 60 without significant comorbidities. In 2018, Everhart et al. [21] published a systematic review of the outcomes of meniscus repair in patients aged 40 years and older. From the 225 articles found in the literature about meniscal repair in adults, only 11 presented outcomes for patients in this age group (148 patients in total). The review demonstrated that the success rates for meniscal repair in this age group were comparable to those of younger patients, as the failure rate among all patients ≥40 years was 10%. The authors, therefore, stated that age, as an independent factor, should not be considered a contraindication for meniscus repair. Among the 148 patients reviewed by Everhart et al., only 23 were treated using the all-inside suture technique. All these cases belonged to an article published by Barrett et al. back in 1998 [22]. Although the authors did not assess patients’ clinical outcomes with scoring systems, only 13% of their cases were symptomatic at their latest follow-up, while the remaining 87% had good clinical outcomes. More recent studies by Zhu et al. [23] and Ventura et al. [24] confirmed low complication rates associated with encouraging functional results. Our results agree with Barrett et al. and the most recent literature, as none of our 23 cases suffered from surgical failures. Our cohort also presented encouraging functional results, as testified by the mean KOOS score that increased from a pre-operative value of 55.1 to a post-operative 87.4. These findings suggest that meniscal repair with all-inside sutures can also be a reliable and effective therapeutic approach for highly demanding and active patients over 40 years of age.

We acknowledge that our study has some limitations. One of them is represented by the retrospective nature of our study, which did not allow the complete standardization of the post-operative follow‐up procedures for each patient. The small size of our cohort represents another limitation, as operating with a broader population would have allowed us to perform more statistical analysis and increase the significance of our findings. These limits could be overcome by performing similar evaluations on a prospective basis and broader populations.

Despite these limitations, our study provides new evidence about the effectiveness of the all-inside meniscal sutures for active patients over 40 with high functional demands who do not suffer from significant comorbidities. Modern meniscal repair techniques can allow a good functional recovery, allowing most treated patients to return to their previous activities of daily living. In modern Western countries, the share of highly demanding patients over 40 is constantly increasing; orthopedic surgeons should extend meniscal repair to these middle-aged patients, who could benefit from the treatment both from a physical and psychological point of view [25,26]. Furthermore, preserving the integrity of the meniscus prevents and delays the degeneration of the cartilage surfaces of the knee, presenting an earlier onset of knee osteoarthritis.

## Conclusions

In conclusion, the all-inside suture technique can represent a suitable and reliable solution for suturable meniscal tears. Preserving the meniscus and providing good functional results allows patients to return to their daily activities and promote their clinical conditions and quality of life in mid- and long-term scenarios.

## References

[1] Jones JC, Burks R, Owens BD, Sturdivant RX, Svoboda SJ, Cameron KL (2012). Incidence and risk factors associated with meniscal injuries among active-duty US military service members. J Athl Train.

[2] Bhan K (2020). Meniscal tears: current understanding, diagnosis, and management. Cureus.

[3] Xu C, Zhao J (2015). A meta-analysis comparing meniscal repair with meniscectomy in the treatment of meniscal tears: the more meniscus, the better outcome?. Knee Surg Sports Traumatol Arthrosc.

[4] Beaufils P, Pujol N (2017). Management of traumatic meniscal tear and degenerative meniscal lesions. Save the meniscus. Orthop Traumatol Surg Res.

[5] Pujol N, Beaufils P (2019). Save the meniscus again!. Knee Surg Sports Traumatol Arthrosc.

[6] Metcalf MH, Barrett GR (2004). Prospective evaluation of 1485 meniscal tear patterns in patients with stable knees. Am J Sports Med.

[7] Matelot D, Schnell F, Kervio G, Ridard C, Thillaye du Boullay N, Wilson M, Carre F (2016). Cardiovascular benefits of endurance training in seniors: 40 is not too late to start. Int J Sports Med.

[8] Peluso MA, Guerra de Andrade LH (2005). Physical activity and mental health: the association between exercise and mood. Clinics (Sao Paulo).

[9] Garrett WE Jr, Swiontkowski MF, Weinstein JN (2006). American Board of Orthopaedic Surgery practice of the orthopaedic surgeon: part-II, certification examination case mix. J Bone Joint Surg Am.

[10] Heckmann TP, Barber-Westin SD, Noyes FR (2006). Meniscal repair and transplantation: indications, techniques, rehabilitation, and clinical outcome. J Orthop Sports Phys Ther.

[11] Scott GA, Jolly BL, Henning CE (1986). Combined posterior incision and arthroscopic intra-articular repair of the meniscus. An examination of factors affecting healing. J Bone Joint Surg Am.

[12] Turman KA, Diduch DR (2008). Meniscal repair: indications and techniques. J Knee Surg.

[13] Buseck MS, Noyes FR (1991). Arthroscopic evaluation of meniscal repairs after anterior cruciate ligament reconstruction and immediate motion. Am J Sports Med.

[14] Eggli S, Wegmüller H, Kosina J, Huckell C, Jakob RP (1995). Long-term results of arthroscopic meniscal repair. An analysis of isolated tears. Am J Sports Med.

[15] Kurosaka M, Yoshiya S, Kuroda R, Matsui N, Yamamoto T, Tanaka J (2002). Repeat tears of repaired menisci after arthroscopic confirmation of healing. J Bone Joint Surg Br.

[16] Bach BR Jr, Dennis M, Balin J, Hayden J (2005). Arthroscopic meniscal repair: analysis of treatment failures. J Knee Surg.

[17] Abrams GD, Frank RM, Gupta AK, Harris JD, McCormick FM, Cole BJ (2013). Trends in meniscus repair and meniscectomy in the United States, 2005-2011. Am J Sports Med.

[18] Lyman S, Hidaka C, Valdez AS (2013). Risk factors for meniscectomy after meniscal repair. Am J Sports Med.

[19] Steadman JR, Matheny LM, Singleton SB, Johnson NS, Rodkey WG, Crespo B, Briggs KK (2015). Meniscus suture repair: minimum 10-year outcomes in patients younger than 40 years compared with patients 40 and older. Am J Sports Med.

[20] Mordecai SC, Al-Hadithy N, Ware HE, Gupte CM (2014). Treatment of meniscal tears: an evidence based approach. World J Orthop.

[21] Everhart JS, Higgins JD, Poland SG, Abouljoud MM, Flanigan DC (2018). Meniscal repair in patients age 40 years and older: a systematic review of 11 studies and 148 patients. Knee.

[22] Barrett GR, Field MH, Treacy SH (1998). Clinical results of meniscus repair in patients 40 years and older. Arthroscopy.

[23] Zhu S, Li X, Lu Z (2023). Arthroscopic repair of degenerative medial meniscus tears in patients aged over 45 years resulted in favorable clinical outcomes and low clinical failure rates at a minimum 2-year follow-up. Knee Surg Sports Traumatol Arthrosc.

[24] Ventura M, Seabra P, Oliveira J (2023). Meniscal injuries in patients aged 40 years or older: a comparative study between meniscal repair and partial meniscectomy. Cureus.

[25] Aggio D, Papacosta O, Lennon L, Whincup P, Wannamethee G, Jefferis BJ (2017). Association between physical activity levels in mid-life with physical activity in old age: a 20-year tracking study in a prospective cohort. BMJ Open.

[26] Pearce M, Garcia L, Abbas A (2022). Association between physical activity and risk of depression: a systematic review and meta-analysis. JAMA Psychiatry.

